# Canine visceral leishmaniasis: Diagnosis and management of the reservoir living among us

**DOI:** 10.1371/journal.pntd.0006082

**Published:** 2018-01-11

**Authors:** Bruno L. Travi, Anabela Cordeiro-da-Silva, Filipe Dantas-Torres, Guadalupe Miró

**Affiliations:** 1 Department of Internal Medicine-Division of Infectious Diseases, University of Texas Medical Branch, Galveston, Texas, United States of America; 2 Instituto de Investigação e Inovação em Saúde, Universidade do Porto, Porto, Portugal; 3 Parasite Disease Group, Instituto de Biologia Molecular e Celular, Universidade do Porto, Porto, Portugal; 4 Departamento de Ciências Biológicas, Faculdade de Farmácia da Universidade do Porto, Porto, Portugal; 5 Department of Immunology, Aggeu Magalhães Institute, Oswaldo Cruz Foundation, Recife, Pernambuco, Brazil; 6 Veterinary Faculty, Animal Health Department, Universidad Complutense de Madrid, Madrid, Spain; Institute of Tropical Medicine, BELGIUM

## Abstract

This article reviews essential topics of canine visceral leishmaniasis (CVL) due to *Leishmania infantum* infection. It focuses on the current serological and molecular diagnostic methods used in epidemiological research and veterinary clinics to diagnose CVL and includes new point-of-care (POC) tests under development. The efficacy of different treatment regimens on the clinical improvement and infectiousness of dogs is also addressed. In the last section, the review provides a critical appraisal of the effectiveness of different control measures that have been implemented to curb disease transmission.

## Author summary

Dogs are the principal reservoir hosts of *L*. *infantum* and consequently play a critical role in the transmission cycle of urban VL, which also affects humans. This review provides updated information on important topics such as diagnostic tests and dog treatments that improve dog health and decrease their transmission efficacy to insect vectors. A critical review of control measures is also provided.

## Introduction

CVL negatively impacts society from medical, veterinary, and societal standpoints. Studies on risk factors for human infection with *L*. *infantum* have yielded opposing results, but a meta-analysis suggested that owners of infected dogs and household members could be at high risk of infection, at least in the Americas [1]. Based on this generalized concept, strategies to comply with public health guidelines typically lead to difficult or expensive decisions. In developed countries, infected dogs are subjected to different treatment protocols that improve the clinical condition but do not clear *L*. *infantum*, while in developing countries, the recommended euthanasia of infected dogs generates societal conflicts [2–4]. Herein, we review current serological and molecular tools for the diagnosis of CVL, the impact of treatment on infectiousness, and control strategies to prevent infection and disease development.

## Methods

The selection of studies referenced in this review was based on searches in PubMed, Library of Congress, Web of Science, Scielo, and Google Scholar, with no specific year range. The search strategy included combinations of the following key words: “canine visceral leishmaniasis,” “leishmaniasis,” “diagnosis,” “molecular diagnostic methods,” “serology,” “treatment,” “xenodiagnosis,” “infectivity,” “prevention,” “control,” “dog culling,” and “vaccination.” Paper selection was grounded in the specific information each article provided to the different sections of this review.

## The utility of standard and newer rapid diagnostic tests in the clinic and in the field

### Molecular diagnosis

The analytical sensitivity of molecular tests suggests they can detect between 0.001 and 0.1 parasite/reaction [5–8]. However, determining the actual diagnostic efficacy at different infection stages could be problematic due to the relatively heterogeneous clinical criteria used in different studies. Before guidelines were established [9], categorization as oligosymptomatic or polysymptomatic animals according to the number of CVL signs and symptoms could vary between studies, making them difficult to compare.

#### Parasite targets

The DNA minicircles contained in *Leishmania* kinetoplast DNA (kDNA) has made this molecular target the basis for the majority of molecular tests aimed at detecting *Leishmania* species, although other targets have been utilized such as Internal Transcribed Spacer-1 (ITS-1), 18S rRNA, and glycoprotein-63 (gp-63) (S1 Table) [10,11]. The specificity of molecular tests is regularly 95% to 100% [8,12–14]. Most reports showed that kDNA-based conventional PCR (cPCR) has a sensitivity of 89% to 100% in blood samples of polysymptomatic dogs [8,15,16]. However, the sensitivity decreases when the method is applied to asymptomatic dogs (e.g., loop-mediated amplification [LAMP], PCR) [5,6]. Real-time quantitative PCR (qPCR) now is considered the most reliable diagnostic method due to its higher sensitivity [8]. Different molecular tests evaluated in the same dog samples (n = 67 dogs) showed that qPCR was more sensitive (91%) than nested kDNA cPCR (72%), nested ITS-1 PCR (54%), OligoC-Test (70%), and PCR hybridization (61%) [8]. Furthermore, evaluations of bone marrow showed that qPCR (TaqMan) detected 0.2 parasites/mL, while cPCR resulted positive only when samples contained >30 parasites/mL [5]. A rapid, low-cost qPCR assay based on a previously published PCR method [5] was optimized by reducing the reaction volumes and DNA amplification time. The test amplified *L*. *infantum* or *L*. *braziliensis* with good sensitivity (0.002 parasite/reaction) [17].

#### Sample source

The sample source has a significant influence on the capacity of molecular tests to identify infected dogs. Bone marrow and lymph node samples yield the highest number of positive results compared with other sites regardless of the clinical status (S1 Table) [18,19].

Less invasive and sensitive diagnostic methods such as swab rubbing of the conjunctiva and oral mucosa or snout provided samples with higher parasite loads than blood [13,18,20,21]. Utilization of ITS-1 cPCR or kDNA PCR hybridization found that 91% to 92% of naturally infected dogs were positive in conjunctival samples [13,22,23].

#### POC molecular tests

POC methods utilize polymerases capable of amplifying DNA at constant temperatures, not requiring sophisticated equipment or a high level of expertise to run the tests. LAMP (Eiken Chemical Company, Tokyo, Japan) uses a DNA polymerase with strand-displacement activity and four primers that recognize six different DNA regions, making it highly specific [24]. This method was previously evaluated in blood of VL patients (*L*. *donovani*) with sensitivities of 90.7% to 96.4% and specificities of 98.5% to 100% [25,26]. In China, the utilization of LAMP in conjunctival samples identified 61% infected dogs, which was similar to cPCR (58.6%) and significantly higher than both microscopy (10.8%) or the proportion of dogs positive by ELISA (40.5%) [6]. However, these kDNA primers designed from an *L*. *infantum* strain isolated in China did not amplify strains from other countries. Further typification of these strains using standard methods is still required.

Another isothermal molecular method for diagnosing multiple pathogens is based on recombinase polymerase amplification (RPA; TwistDx, UK). The RPA mixture contains three core proteins: recombinase, single-strand DNA binding protein, and strand-displacing polymerase. In combination with specific forward and reverse primers, it enables DNA amplification at constant temperatures between 24°C and 45°C and 15- to 60-minute incubation times depending on the pathogen to be detected [27]. The amplification products can be read with the naked eye in a lateral flow strip. An RPA-lateral flow test (RPA-LF) for CVL was recently developed based on primers and probe that target *L*. *infantum* kDNA minicircles [7]. This qualitative test is run at 42°C for 40 minutes and subsequently read in a lateral flow strip (Milenia Hybridetect, TwistDX or UStar Biotechnologies, Hangzhou, China). It has an analytical sensitivity similar to qPCR (SYBR Green) and can detect 40 parasites per mL of blood. RPA-LF detected *L*. *infantum* in the oral mucosa of symptomatic dogs. Screening of 30 clinically normal dogs from an endemic area in Argentina showed that 50% of the animals were subclinically infected as determined by RPA-LF compared with 13.3% that were serologically positive using recombinant kinesin-39 (rK39) test [7].

LAMP and RPA quantitative diagnostic methods that were developed for other pathogens [28–31] are not applicable as POC tests because they require relatively costly equipment and trained personnel.

### Serological diagnosis

The seroconversion to parasite antigens can be as early as one month after an infective phlebotomine bite [32]. Active CVL is usually associated with significant antibody titers of all classes, whereas low antibody levels are characteristic of subclinical infections or exposed but uninfected dogs [33]. Although the use of different antibody isotypes was proposed for improving serological evaluations [34–36], the most recent data from a cohort of 134 dogs suggest that isotype responses have no major predictive value [37]. Increased levels of immunoglobulin G2 (IgG2) were associated with protective responses, while rising IgG1 production was considered as bad prognosis [35]. IgE and IgA seem to be detected mostly in active CVL but with low predictive value in asymptomatic dogs [35,38,39].

Clinical and epidemiological studies have used serological tests involving whole parasites, soluble parasite extracts, or recombinant proteins derived from genes of interest. The recent development of chimeric antigens with relevant protein epitopes demonstrated the capacity of this method to detect specific antibodies during active disease or asymptomatic infection [40,41].

The direct agglutination test (DAT) is based on the agglutination of trypsinized Coomassie-stained *Leishmania* promastigotes by anti-*Leishmania* antibodies. It was the first serological test developed for field use. It is simple, cheap, and reliable, with proven clinical accuracy (S1 Table) [42,43]. Moreover, it can be performed in laboratories not requiring electrical equipment and has up to 2 years of shelf life. DAT has long incubation times and requires some level of expertise to run and read the test [43,44]. It has a sensitivity and specificity of 91% to 100% and 72% to 100%, respectively, yet subjective reading of end-point titers leads to interobserver discrepancy [43,45]. Despite those drawbacks, DAT is well accepted as a routine serologic test usually applied to a large number of samples [46]. The fast agglutination screening test (FAST) is a modification of DAT based on a single serum dilution above the cutoff point of normal sera. It requires shorter incubation times and has been optimized for screening large dog populations [46].

The immunofluorescence antibody test (IFAT) against *Leishmania* promastigotes is the reference qualitative serological method for CVL diagnosis [47]. The use of IFAT is restricted to laboratory settings because it needs specialized equipment and trained personnel [48]. The specificity and sensitivity are close to 100% in symptomatic animals. Some notable limitations are the cross-reactivity with other pathogens such as trypanosomes [47,48] and the significantly lower sensitivity for identifying asymptomatic dogs compared with ELISA [41].

ELISA allows screening large numbers of samples utilizing antigen-coated microplates and a spectrophotometer that determines antibody titers by optical density. The potential for absolute antibody quantification renders ELISA as a powerful tool that is less susceptible to operator bias. One of its strengths is the possibility of using combinations of multiple antigens, thereby increasing the sensitivity and/or specificity of the method (S2 Table) [49,50].

Flow cytometry (FC) is an emerging technology [51] that quantifies antibodies against *Leishmania* surface antigens, avoiding cross-reactivity against more conserved intracellular structures. Using amastigotes or promastigotes, this method achieved high levels of sensitivity and specificity and has been shown to distinguish serological profiles of infected but clinically healthy versus sick dogs [52].

#### Antigens used for serodiagnosis

Methods that use crude promastigote antigens are highly sensitive for detecting subclinical and clinical infections but have lower specificity than methods that employ other antigens [44,53]. Soluble antigens from axenic amastigotes were equivalent to promastigotes when used as crude lysates [52]. Recombinant proteins are considered good candidate antigens for field diagnosis because they are easily adsorbed on several scaffold surfaces and yield reproducible results [49,54–56]. Among them, the most successful antigen moving from academic to clinical settings was rK39. This 39–amino acid repeat of a kinesin-related protein is highly conserved among viscerotropic *Leishmania* species [41,44]. It has good capacity to detect active CVL, demonstrating high sensitivity (90%–100%) in canine cohorts from the Mediterranean basin and South America [57–60]. Notwithstanding, K39 was less specific and sensitive than other serological methods for detecting asymptomatic infections [49,57]. Fluctuations in sensitivity and specificity were also described when applied to human VL [61]. In Brazil, the K39 dipstick test had suboptimal specificity (less than 80%) when compared to conventional promastigote ELISA [62]. The variability of K39 efficacy according to endemic regions prompted the search for better alternatives to evaluate active CVL and asymptomatic infection. For instance, rKLO8 and rK26 were proposed as antigens capable of increasing the diagnostic accuracy of CVL [63]. The combination of these two antigens exhibited higher sensitivity (85%) and specificity (93%) compared with their individual sensitivity and specificity (KLO8, 68% and 92%, respectively; rK26, 77% and 91%, respectively). Also, chimeric multi-epitope proteins are being engineered to improve the detection of asymptomatic infected dogs [64]. Novel approaches like immunoproteomics identified an uncharacterized hypothetical protein with high specificity and sensitivity for CVL [65]. This hypothetical protein used in an ELISA format showed 100% sensitivity and specificity in samples of sick and clinically healthy dogs infected with *L*. *infantum*. Also, *L*. *infantum* secreted proteins or multi-antigen printing immunoassay (MAPIA) of recombinant proteins were proposed as sources of antigens for diagnosis [66–68]. In a recent study, the utilization of excreted-secreted antigens (proteins 26.5 to 31.5 kDa) from *L*. *infantum* applied to ELISA (ESA-ELISA) and ESA-blot formats detected 100% of infected dogs [68].

POC serological tests are based principally on qualitative immunochromatographic tests that are read with the naked eye. They obviate the quantitative assessment of the immune response in favor of field applicability and user-friendly design. Most commercial kits currently available use a few validated recombinant antigens like rK39, rK26, and rKE16 [69,70]. Distinct biological fluids such as plasma, serum, whole blood, or blood adsorbed onto filter paper can be used in most of the tests. The specificity of commercial kits is usually high (>90%), as opposed to the sensitivity, which can be highly variable (30%–90%) and a matter of concern for both clinical and epidemiological applications [41,62,70–72].

## Impact of treatment on dog infectiousness

In Europe, the treatment of CVL has been almost exclusively limited to the use of pentavalent antimony meglumine antimoniate. The recommended regimen of 35 to 50 mg/kg subcutaneously twice daily for 4 to 6 weeks [9] demonstrated good clinical efficacy but without clearing the infection.

The combination with allopurinol showed better response to treatment of sick dogs, with good clinical recovery and improvement of hematological and biochemical abnormalities [73]. Allopurinol administration (10 mg/kg by mouth twice daily) for 6 to 12 months after an antimonial course was highly leishmaniostatic, maintaining treated dogs in long-term clinical remission [74].

Other leishmanicidal drugs such as miltefosine at a dosage of 2 mg/kg by mouth once daily for 4 weeks, in combination with allopurinol, demonstrated leishmanicidal efficacy in naturally infected dogs [75]. Other drugs against CVL have been studied in vivo or in vitro such as aminosidine, pentamidine, enrofloxacine, and marbofloxacine, but further controlled clinical trials are needed [76,77]. Some of them might be used as an alternative when first line therapy fails or renal function is altered [78]. A new therapeutic trend is the combination of parasiticidal–parasitostatic drugs and immunomodulators aimed at reducing parasite burden and establishing an appropriate immune response (domperidone or Protein Aggregate Magnesium-Ammonium Phospholinoleate-Palmitoleate Anhydride (P-MAPA) [79,80].

Nevertheless, most dogs remain infected and might relapse, becoming infectious to healthy dogs and other hosts, including humans. Until new therapies that consistently clear *L*. *infantum* are found, dog infectivity can be managed by applying topical repellents that can reduce transmission risks to near zero (see section “Are prevention and control strategies working?”).

Xenodiagnosis (sand fly feeding on host) is the best alternative to determine dog infectiousness, but this method can only be applied in specialized research centers [81]. The first research using posttreatment xenodiagnosis that demonstrated significant reduction of dog infectiousness was published by Gradoni et al. [82]. Afterwards, Alvar et al. [83] evaluated six dogs treated with meglumine antimoniate in combination with allopurinol, reporting that all dogs were noninfective for a “few months” after chemotherapy. Guarga et al. [84] evaluated dogs (n = 10) treated with meglumine antimoniate and found a significant reduction in dog infectivity, which persisted until the end of the study (120–180 days). Another study used *Lutzomyia longipalpis* to evaluate the effectiveness of a liposome formulation of meglumine antimoniate [85]. Dogs were treated parenterally with antimonials, empty liposomes, or isotonic saline solution. A significant reduction of dog infectivity was found in the group treated with antimonials as determined 150 days post treatment. More recently, Miró et al. [86] evaluated 32 dogs with CVL that were subjected to three different treatments: antimonials, antimonials plus allopurinol, or allopurinol alone. They showed a considerable reduction of infectivity of all three groups and significant decrease of parasite burden in bone narrow. A study conducted in Brazil by da Silva et al. [87] included 52 dogs distributed in six treatment groups: liposomal formulation of antimonials, allopurinol, liposomal formulation of antimonials plus allopurinol, empty liposomes plus allopurinol, empty liposomes, and saline solution. The negative xenodiagnosis and qPCR quantification of *L*. *infantum* in the skin below the infectious threshold indicated that antimonials plus allopurinol was the most efficient regime to decrease infectivity to sand flies.

A recent study in Brazil (n = 36 dogs) assessed the infectivity of sick dogs after a conventional miltefosine treatment. After three months of treatment, there was a significant reduction of parasite load in the bone marrow, lymph nodes, and skin. These results correlated with the xenodiagnoses in which 74.2% of dogs were noninfectious for sand flies [88].

In conclusion, treatment of sick dogs in endemic areas decreases canine infectiousness, thus diminishing the epidemiological risks for humans and other uninfected dogs. Assessment of parasite loads in the ear skin by qPCR has been proposed as surrogate marker of infectiousness [89] and may be used whenever xenodiagnosis is not available. Nevertheless, further studies on posttreatment infectiousness of canines using different drugs are still needed. Despite not being a simple method, xenodiagnosis is a useful tool to assess the infectious capacity of dogs treated with new drugs and/or new treatment regimens.

## Are prevention and control strategies working?

Several strategies have been proposed for preventing and controlling CVL at both individual and population levels. Prevention of infection can be achieved by applying insecticide-impregnated collars or spot-on products (e.g., deltamethrin, permethrin, flumethrin, fipronil) on dogs, whereas the risk of disease development can be reduced by vaccination or immunomodulation. The effectiveness of some of these strategies has been assessed by recent systematic reviews and meta-analyses [90,91]. The conclusions based on either parasitological or serological evidence were that repellents and prophylactic medication (i.e., domperidone) tended to reduce the proportion of dogs infected with *L*. *infantum* [90]. Nonetheless, Wylie et al. [91] also concluded that well-designed, adequately powered, and properly reported randomized clinical trials are needed to clearly establish the efficacy of vaccines against CVL.

Culling of infected dogs has been recommended as a control strategy in many endemic countries. A cluster-randomized trial in Northeast Brazil reported a low to moderate effectiveness of dog culling and concluded that there is an urgent need for revision of the Brazilian VL control program [92]. González et al. [93] reviewed two trials from Brazil that evaluated the effects of culling infected dogs compared to no intervention or indoor residual spraying. Although these trials reported a reduction in seroconversion over 18-month follow-up, they did not measure or report effects on clinical disease in humans [93]. Mathematical models suggest that dog culling alone is not effective in areas of high transmission [94]. According to Costa et al. [94], the indiscriminate culling of healthy, seropositive dogs may jeopardize the effectiveness of the control program if low specificity tests are used, thus increasing the chance of generating outrage in the population and reducing the adherence to the program. In Iran, the utilization of insecticide-impregnated dog collars in nine villages showed significantly decreased seroconversion in children (odds ratio [OR] 0.57; 95% CI 0.36–0.90; *p* = 0.017) and dogs (OR 0.46; CI 0.30–0.70; *p* = 0.0003) compared with nine nonintervened villages [95].

While the above-mentioned strategies (e.g., repellents, vaccination, and immunomodulation) may work at the individual level, their effectiveness at the population level still needs to be demonstrated when the intervention is transferred to the communities. Indeed, the effectiveness of strategies like community-wide application of insecticide-impregnated collars is directly dependant on coverage and loss rate [96]. This is particularly unrealistic in developing countries, considering the limited economic and human resources, especially in periods of crisis and political upheavals. Moreover, in these countries, stray dogs will not be targeted by such strategies and may function as infection reservoirs. As for any zoonosis, stray dog population management should be part of any VL control programs.

## Conclusion

Molecular tools are mainly restricted to research laboratories, but progress is being made towards field applicability. The costs of real-time PCR machine and the need for sophisticated laboratory infrastructure highlight the importance of validating and implementing POC molecular tests that could be used in-clinic and in the field. The veterinarians should rely also on complementary serological information to make the best decision regarding both the animal’s health and the epidemiological risk it entails. The remaining challenge for serological tests is to improve the capacity to differentiate clinically healthy but infected and vaccinated dogs. The evaluation of new antileishmanial drugs should be complemented by standardized follow-up that includes the infectiousness status of dogs at different times post treatment. Nowadays, veterinarians and dog owners have different options to decrease infection risks, of which repellents are still the most important prevention tools as demonstrated by laboratory and field studies. New vaccines that could reduce the risk of disease development and infectiousness of vaccinated dogs are urgently needed.

**Key learning points**

- Understand the practical value that serological and molecular tests have to diagnose CVL in the field or laboratory.
- Become aware of new approaches still under development to diagnose CVL.
- Appreciate the value of treatment to decrease the infectivity of dogs to sand fly vectors of VL.
- Evaluate the efficacy of prevention and control strategies to interrupt transmission of *L*. *infantum* from dogs to sand fly vectors.

**Top five papers**

1. Castellanos-Gonzalez A, Saldarriaga OA, Tartaglino L, Gacek R, Temple E, et al. (2015) A Novel Molecular Test to Diagnose Canine Visceral Leishmaniasis at the Point of Care. Am J Trop Med Hyg 93: 970-975.
2. Sousa S, Cardoso L, Reed SG, Reis AB, Martins-Filho OA, Silvestre R, Cordeiro da Silva A. Development of a fluorescent based immunosensor for the serodiagosis of canine leishmaniosis combining immunomagnetic separation and flow cytometry. PLoS Negl Trop Dis. 2013 Aug 22;7(8):e2371. doi: 10.1371/journal.pntd.0002371. eCollection 2013.
3. da Silva SM, Amorim IF, Ribeiro RR, Azevedo EG, Demicheli C, et al. (2012) Efficacy of combined therapy with liposome-encapsulated meglumine antimoniate and allopurinol in treatment of canine visceral leishmaniasis. Antimicrob Agents Chemother 56: 2858-2867.
4. Costa DN, Codeco CT, Silva MA, Werneck GL (2013) Culling dogs in scenarios of imperfect control: realistic impact on the prevalence of canine visceral leishmaniasis. PLoS Negl Trop Dis Aug 8;7(8):e2355. doi:10.1371/journal.pntd.0002355. eCollection 2013.
5. Werneck GL, Costa CH, de Carvalho FA, Pires e Cruz Mdo S, Maguire JH, et al. (2014) Effectiveness of insecticide spraying and culling of dogs on the incidence of Leishmania infantum infection in humans: a cluster randomized trial in Teresina, Brazil. PLoS Negl Trop Dis Oct 30;8(10):e3172. doi: 10.1371/journal.pntd.0003172. eCollection 2014

## Supporting information

S1 Table Molecular tests for diagnosis of canine visceral leishmaniasis. (DOCX)

S2 Table Serological tests for diagnosis of canine visceral leishmaniasis. (DOCX)
