# Supplementary material for: Canine visceral leishmaniasis: Diagnosis and management of the reservoir living among us
Source: PLoS Negl Trop Dis. 2018 Jan 11;12(1):e0006082. doi: 10.1371/journal.pntd.0006082 (PMC5764232; doi:10.1371/journal.pntd.0006082)
Supplement: S1 Table — (DOCX) [file pntd.0006082.s001.docx]

**Supplementary Table 1. Molecular tests for diagnosis of canine visceral leishmaniasis.**

| **Reference** | **Extraction Method** | **Parasite Target** | **Test** | **Main Objective** | **Sensitivity**  **(analytical or diagnostic)** | **Specifi- city** | **Sample type[s]** | **Dog number per group** |
| --- | --- | --- | --- | --- | --- | --- | --- | --- |
| Lachaud et al., 2002 | Phen-Chlor | rRNA,  kDNA | cPCR, PCR-hyb. | Evaluate multiple primers | Symp. 100% | 100% | Blood | 17 symp, serol+  7 asymp,serol+ |
| Lachaud et al., 2002 | prot K, Phen-Chlor of buffy coat | kDNA, 18s rRNA | cPCR, PCR-hyb. | Evaluate test for surveillance | Symp:  Blood 89.4%  Skin 75.7%,  Asymp:  Blood 65.2%  Skin 44.9% | 100% | Blood, skin, BM, LN, dermal scrapings | 307 dogs; varied numbers per sample type |
| Manna et al., 2004 | prot K, Phen-Chlor | kDNA | cPCR | Evaluate sensitivity of sampling sites; 12, 24 mo follow up | Symp:  Blood 94%  LN 98.9%  Skin 94.7% | ND | Blood, LN, skin biopsy | 95 symp |
| Francino et al., 2006 | PK buffer, prot K  Leukocyte pellet | kDNA | cPCR vs. qPCR | Evaluate the advantage of real time PCR | Taq qPCR:  BM: 0.2 ps/mL to 6,8x10^5^ ps/mL  cPCR: >30 ps/mL | ND | Blood, BM | 44 symp; varied numbers per sample type |
| De Almeida Ferreira et al., 2008 | Phen-Chlor | kDNA | cPCR, PCR-hyb. | Evaluate conjunctival swab sampling | cPCR: 73.9% conjunctiva  13% buffy coat  PCR-hyb: 91.3% conjunctiva  21.7% buffy coat | ND | Conjunctiva, buffy coat | 23 symp. serol+ |
| Carson et al., 2010 | Phen-Chlor | kDNA,  ITS-1 rRNA | cPCR, | Compare OligoC-TesT with other PCRs | Polysymp:  OligoC-TesT 93%  cPCR (kDNA) 97%  PCR-hyb 83%  nested-PCR (ITS-1) 90%  qPCR 100%  Asymp:  OligoC-TesT 61%  stPCR (kDNA) 62%  PCR-hyb 54%  nested-PCR (ITS-1) 42%  qPCR 88% | 100% for all tests | BM | 67 infected |
| De Melo et al., 2010 | Prot K; phen-chlor | kDNA, ITS-1 | PCR-hyb.,  nPCR[ITS-1] | Evaluate conjunctival swab sensitivity | PCR-hyb:  Conjunctiva 90%  Skin biop 57%  Blood 13%  nPCR:  Conjunctiva 83%  Skin biop 50%  Blood 57% | 100% | Blood, conj, skin biopsy | 30 infected asymp. |
| Lombardo et al., 2012 | Prot K, Purelink kit | kDNA | qPCR | Evaluate conjunctival swab sensitivity | LN 25%  Conj. 22%  Oral 9%  Blood 6% | ND | LN, Conjunctiva., oral, blood | 163; outdoor kennels. Mixed infection status |
| Almeida et al., 2013 | Prot K Phen-chlor | kDNA | cPCR | Identify the best samples for using PCR | Sympt:  Buf coat 7%  BM 14%  LN 24%  Skin 14%  Ulcer 12% | ND | Blood, BM, LN  Skin, ulcer | 430:  238 asymp, 42 symp, 22 cutaneous ulcers, 150 oligosymp. |
| Ferreira et al., 2013 | Prot K, phen-chlor | kDNA | cPCR | Evaluate non-invasive samples | Nasal 87%  Conj 76%  Skin 81%  BM 90% | 100% | Conjunctiva., nasal, BM, skin biopsy | 62 infected |
| Courtenay et al., 2o14 | Phen-chlor for BM; Qiagen DNeasy for skin | kDNA | qPCR | Correlate dog parasite burden and infectivity to sand flies | BM >10^6^  Ear skin ≥10^4^ | NA | BM, skin | 82 infected (BM)  64 infected (skin) |
| de Sousa Goncalves et al., 2016 | Prot K. Qiagen (hair) and Invitrogen (spleen) | kDNA | cPCR | Evaluate non-invasive sampling (hair)  and determine infectiousness | Hair 80%  Spleen 100% | NA | Hair, spleen aspirate | 15 infected  8 healthy |
| Aschar et al., 2016 | Nucleospin Tissue kit (Oral swab, Conj swab, LN); Illustra Blood Genomic Mini prep(blood) | kDNA | qPCR | Evaluate non-invasive sampling | Symp:  Oral swab 82%  Conjunctival swab 84%  Blood 48%  Asymp:  Oral swab 37%  Conjunctival swab 37%  Blood 60% | NA | Blood, oral swab,  Conjunctival swab,  LN aspirate | 62 symp  30 asymp infect |
| Gao et al., 2015 | Phenol-chlor | kDNA | LAMP[1] | Evaluate non-invasive sampling. Screening clinically normal dogs | LAMP 61.3%  PCR 58.6%  ELISA 40.5% | 100% | Conjunctival swab | 111 asymp.  33 neg. controls |
| Castellanos-Gonzalez et al., 2015 | Qiagen DNeasy | kDNA | RPA-LF[2] | Evaluate a novel isothermal amplification method. Screening clinically normal dogs | RPA-LF 50%  K39 13.3% | 100% | Blood (asymp.)  Oral swab (polysymp.) | 30 asymp. from endemic area  7 polysymp. |

DNA extraction methods: Phen-Chlor: Phenol-Chloroform; Commercial kits: Qiagen; Illustra. prot K: proteinase K. DNA amplification methods: cPCR: Conventional PCR; qPCR: quantitative PCR; nPCR: nested PCR; PCR-hyb: PCR hybridization; LAMP: Loop mediated amplification; RPA-LF: Recombinant polymerase amplification –Lateral Flow. Samples: BM: bone marrow; LN: lymph node; Clinical status: asymp.: asymptomatic; polysymp.: polysymptomatic; symp.: symptomatic
