# Supplementary material for: Canine visceral leishmaniasis: Diagnosis and management of the reservoir living among us
Source: PLoS Negl Trop Dis. 2018 Jan 11;12(1):e0006082. doi: 10.1371/journal.pntd.0006082 (PMC5764232; doi:10.1371/journal.pntd.0006082)
Supplement: S2 Table — (DOCX) [file pntd.0006082.s002.docx]

**Supplementary Table 2. Serological tests for diagnosis of canine visceral leishmaniasis**

| **Reference** | **Antigen** | **Test** | **Main Objective** | **Sensitivity** | **Specificity** | **Limitations** |
| --- | --- | --- | --- | --- | --- | --- |
| Adams et al., 2012 | *Leishmania* promastigote crude lysates | DAT | Evaluate serological status | 72-100% | 91-100% | Discrepancy in routine results |
| Paltrinieri et al, 2016; Swets et al, 1988* | Fixed  *Leishmania* promastigotes | IFAT | Evaluate qualitative serological method | 90% for symptomatic  29.4% for asymptomatic | 100% | Specialized equipment and personal training. |
| Sousa et al., 2013 | Antigen-Coated microspheres (rK39 and LicTXNPx) | FC | Detection of clinical and subclinical forms of CVL | 95.2% | 97.2% | Specialized equipment and personal training |
| Santarem et al., 2010; | rK39 with LinTXNPx; | ELISA | Screening of large numbers of serum samples | 87.1% (96.4% for symptomatic cases and 73.7% for asymptomatic) | 96.3-99% | Require spectrophotometer and antigen coated microplates. |
| Soto et al., 1998. | Multiple-epitope proteins | ELISA | Screening of large numbers of serum samples | 79 to 93% | 96 to 100% | Require spectrophotometer and antigen coated microplates. |
| Solano-Gallego et al., 2014 | *L. infantum* soluble extract | ELISA  IDScreen | Screening of large numbers of serum samples  Evaluate serological studies.  Compare ID Screen with IFAT | ID Screen 95.3%  IFAT 86.9% % | ID screen 100%  IFAT 91.7% | Cross reactivity |
| Solano-Gallego et al., 2014;  Rodríguez-Cortés et al, 2013 | *L. infantum* soluble extract | ELISA  Leiscan | Compare Leiscan with IFAT | Leiscan  92.5%  98%  IFAT  86.9%  65% | Leiscan  100%  100%  IFAT  91.7 %  98.0% | Cross reactivity |
| Solano-Gallego et al., 2014 | *L. infantum* soluble extract | ELISA  Leishmania 96 | Compare efficacy of ID Screen, IFAT and Speed Leish K | ID Screen 95.3%  IFAT 86.9%  Speed Leish K 63.6% | ID Screen 100%  IFAT 91.7%  Speed Leish K 100% | Not available |
| Solano-Gallego et al., 2014 | Kinesin antigens | Membrane ImmunochromatographySpeed Leish K | Evaluate.  Speed Leish K | 63.6% | 100% | Cross reactivity; Only qualitative information |
| Athanasiou et al, 2014;  Rodríguez-  Cortés et al, 2013 | *L. infantum* soluble extract | ELISA  Snap kit | Compare Snap kit with IFAT | Snap kit  89.2%  66%  IFAT  91.1%  65.0% | Snap kit  100%  100%.  IFAT  99.2%  98.0% | Only qualitative information |
| Athanasiou et al, 2014 | Canine *L. infantum* antigen | Membrane Immunochromatography  ImmunoRun kit | Serological studies.  Compare Immunochromatography with IFAT | IFAT  91.1%  ImmunoRun kit 86.2% | IFAT  99.2%  ImmunoRun kit  100% | Only qualitative information |
| Rodríguez-Cortés et al, 2013 | *L. infantum* soluble extract | ELISA indirect INGEZIM® LEISHMANIA | Compare INGEZIM LEISHMANIA with IFAT | INGEZIM LEISHMANIA 78%  IFAT 65.0% | INGEZIM LEISHMANIA 100%  IFAT 98.0% | Not available |
| Rodríguez-Cortés et al, 2013 | *L. infantum* soluble extract | ELISA  INGEZIM® LEISHMANIA VET | Evaluate INGEZIM and LEISHMANIA VET | INGEZIM 78%  LEISHMANIA VET 76.0% | INGEZIM 100%  LEISHMANIA VET 100% | Not available |
| Rodríguez-Cortés et al, 2013 | *L. infantum* soluble extract | Membrane Immunochromatography  INGEZIM® LEISHMACROM | Serological studies[.  Compare INGEZIMLEISHMACROM with Leiscan Leishmania ELISA test | INGEZIM LEISHMACROM 75%  Leiscan Leishmania ELISA test 98% | INGEZIM LEISHMACROM 100%  Leiscan Leishmania ELISA test 100% | Only qualitative information |
| Rodríguez-Cortés et al, 2013 | *Leishmania* antigen sensitized- gold particles | Membrane Immunochromatography  WITNESS® Leishmania | Serological studies.  Compare WITNESSLeishmania with IFAT | WITNESS Leishmania 58%  IFAT 65.0% | WITNESS Leishmania 100%  IFAT 98.0% | Only qualitative information |
| Reithinger et al., 2002; Babakhan et al,, 2009 | rK39 | rK39 dipstick | Evaluate rK39 in mass-screening surveys | 72 to 77% | 61 to 75% | Low sensitivity |
| Akhoundi et al., 2010 | crude lysate of *L. infantum* promastigotes | Direct agglutination test  FAST | Screening surveys Compare DAT with FAST | DAT 89.3-98.1%  FAST 95.40% | DAT 89.4-71.6%  FAST 88.5% | Low sensitivity |

Tests: DAT: Direct agglutination test; IFAT: Indirect fluorescent antibody test; ELISA: Enzyme linked immunosorbent assay;

FC: Flow cytometry; FAST: Fast agglutination screening test. * except in areas endemic for the New World parasite Trypanosoma cruzi that may give false-positive results
